# Behavioral Inhibition Underlies the Link Between Interoceptive Sensitivity and Anxiety-Related Temperamental Traits

**DOI:** 10.3389/fpsyg.2018.01026

**Published:** 2018-06-21

**Authors:** Pessi Lyyra, Tiina Parviainen

**Affiliations:** Department of Psychology, Centre for Interdisciplinary Brain Research, University of Jyväskylä, Jyväskylä, Finland

**Keywords:** interoception, behavioral inhibition, temperament, personality, heart rate

## Abstract

Interoceptive sensitivity (IS) is a biologically determined, constitutional trait of an individual. High IS has been often associated with proneness to anxiety. This association has been explained by elevated autonomic responsiveness in anxious individuals. However, in a heartbeat discrimination task (discrimination of heartbeats’ simultaneity to an external stimulus) low cardiac responsiveness has accompanied enhanced performance. The relation between these factors seems task dependent, and cannot comprehensively explain the link between IS and anxiety. We explored for additional explanatory factors for this link. More specifically, we studied which anxiety-related temperamental traits most strongly predict IS in the discrimination task. Compatibly with earlier findings, IS was positively associated with individual trait anxiety and also other related traits such as negative affect, emotional intensity, and introversion. Interestingly, behavioral inhibition was the temperamental trait that most strongly predicted high IS, and, in fact, accounted for its significant associations with the other anxiety-related temperamental traits. Good performance on heartbeat discrimination task may reflect adaptive attentional control abilities in behaviorally inhibited individuals. These results can improve our understanding of how IS and other traits together determine the personality and wellbeing of a human individual.

## Introduction

Interoception conveys multimodal information from the entire body of a human individual, notably from his/her visceral and cutaneous sensory receptors (e.g., Cameron, 2002; Craig, 2014). The central function of the interoceptive system is in maintaining an individual’s autonomic homeostasis and allostasis (Craig, 2002, 2003, 2009; Barrett et al., 2016; Kleckner et al., 2017). Interoception also contributes to the emergence of affective states (Critchley and Garfinkel, 2017), representation of the self (Seth, 2013), and even processing of stimulus saliency (Menon and Uddin, 2010). The regulation of the body by the interoceptive system operates non-consciously, but integrated sensory information about the general condition of the body also reaches conscious and cortical levels. An interoceptive network comprising of both cortical (e.g., insular, cingulate and orbitofrontal cortices) and subcortical brain regions (e.g., hypothalamus, periaqueductal gray, parabrachial nucleus, and nucleus of the solitary tract) has been identified (e.g., Craig, 2014; Kleckner et al., 2017). Moreover, processing of interoceptive information is integral to the central large-scale functional brain networks, the ‘salience network’ and the ‘default mode network’ (Kleckner et al., 2017). In other words, interoception is suggested to play a critical role in both subjective wellbeing and processing of external stimuli.

Conscious discrimination of interoceptive signals in humans is studied mostly by experiments applying heartbeat detection tasks. Participants typically either count the number of their own heartbeats across a given temporal span (tracking task; Schandry, 1981) or discriminate whether their heartbeats are simultaneous or not to an external stimulus (discrimination task; Whitehead et al., 1977). Both tasks have proven reliable and intercorrelated (e.g., Knoll and Hodapp, 1992; Schulz et al., 2013; Garfinkel et al., 2015; for criticisms of these traditional tasks, see, e.g., Kleckner et al., 2015; Brener and Ring, 2016). They are held to reflect how accurately an individual perceives the states of one’s autonomous nervous system (Herbert et al., 2012; Garfinkel et al., 2015), referred to here as ‘interoceptive sensitivity (IS).’

The interoceptive system also is likely to contribute to the personality of a human individual. Similarly to temperament, construed as the early appearing or innate individual differences in the threshold, intensity, duration and predictive regulation of responses to external or internal stimuli (Rothbart, 2011), IS can be seen as one of the constitutional individual traits, defined by Garfinkel and Critchley (2013) as those ‘relatively stable across the life span.’ A pivotal further issue is how high sensitivity to interoceptive signals links with other individual constitutional traits (Garfinkel and Critchley, 2013), particularly anxiety (for a review, see Domschke et al., 2010; Paulus and Stein, 2010), also as a trait (e.g., Pollatos et al., 2009). This link has been explained by that the high responsiveness of autonomic nervous system may sensitize the anxious individual to signals from within his/her body (Herbert et al., 2007; Pollatos et al., 2007). Relatedly, emotional intensity has been found to accompany IS (e.g., Wiens et al., 2000).

Neural studies have reported that increased right insular activity mediates the relationship between bodily sensibility and anxiety (Terasawa et al., 2013). Activation of the interoceptive brain network in the right hemisphere is suggested to underpin avoidance motivation, i.e., sensitivity to – even anticipated – negative outcomes, manifested as behavioral inhibition and increased anxiety (Svihra and Katzman, 2004; Craig, 2005, 2009, 2014; Muris et al., 2011). To our knowledge, nevertheless, the association between avoidance motivation or behavioral inhibition and IS has not been directly tested.

Interestingly, however, the results concerning autonomic responsiveness and heartbeat detection have been mixed; some studies reported poorer heartbeat detection in connection with increased responsiveness (Knapp-Kline and Kline, 2005; Fairclough and Goodwin, 2007; Schulz et al., 2013), or no association between these variables (Yoris et al., 2015). The association may thus be task dependent (for studies comparing the tasks, see, e.g., Schulz et al., 2013; Garfinkel et al., 2015). If high autonomic responsiveness cannot account for improved performance on the discrimination task – nor the latter’s relation to anxiety – it is important to explore for factors underlying the relation between IS and anxiety-related traits in that paradigm.

We studied in a normal healthy population the association between IS in the discrimination task and individual temperament. Specifically, to add to the existing literature, we aimed to reveal which temperamental measures are most relevant for IS. We expected that individual differences in temperamental traits related to anxiety and avoidance motivation – particularly behavioral inhibition but also introversion, temperamental anxiety, negative affect, and emotional intensity – collectively predict IS.

## Materials and Methods

### Participants

Fifty (23 male, *M_age_* = 24.46, *SD* = 3.89) healthy university students, recruited using mailing lists and notices on campus bulletin boards, participated in this study as a part of a larger magnetoencephalography (MEG) study (not reported here). All participants had previous experience with a similar heartbeat detection task. They completed questionnaires measuring temperament and personality traits, emotion intensity as well as physiological background information (body mass index [BMI]).

This study was carried out in accordance with the recommendations of American Psychological Association (APA) and the Declaration of Helsinki, with written informed consent from all subjects. The protocol was approved by The Ethical Committee of the University of Jyväskylä.

### Stimuli and Procedure

On arrival, participants were prepared for MEG and ECG measurements, instructed about the experimental task and seated in a chair. The stimuli were presented on a screen with a distance of 105 cm from the subject.

**FIGURE 1:**
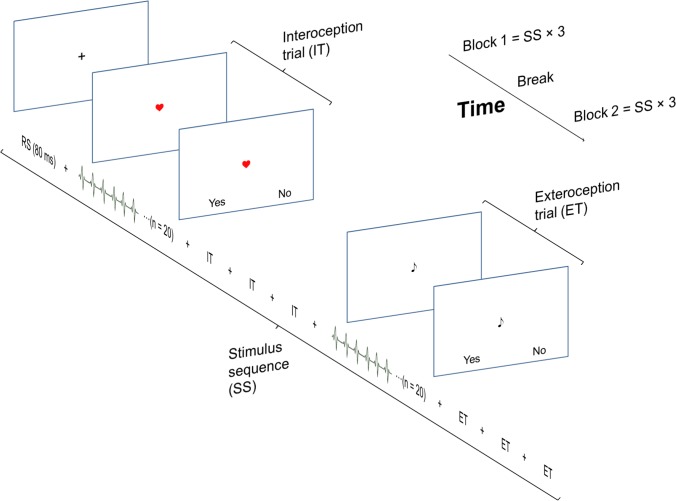
The experimental task consisted of two identical blocks, each consisting of 3 stimulus sequences (SS). A SS started with 4 interoception trials followed by 4 exteroception trials, resulting in total number of 24 interoception and 24 exteroception trials. Each trial consisted of twenty heartbeats. The SS were separated by an 80 s break/resting state (RS). The presentation of the simultaneity/non-simultaneity and the deviance/non-deviance condition trials were randomized across a block.

The experimental procedure is illustrated and explained in **Figure 1**. Participants performed a heartbeat and an auditory discrimination task (a control condition for MEG, not analyzed here). In both tasks, participants were presented with 96 trials, each consisting of a sequence of 20 auditory stimuli presented through earphones with an individually adjusted volume. The auditory stimuli were locked to the participant’s heartbeats either simultaneously (simultaneity condition, 50%), or with a delay of 40% of the duration of the previous inter-beat interval (non-simultaneity condition, 50%). The auditory stimulus was an 800 Hz, 100 ms tone (non-deviance condition), but on half of the trials, one slightly deviant 785 Hz, 100 ms target tone was randomly interspersed between the other tones (deviance condition). Independent of the task, the stimulus sequences (SS) were identical except for the duration of the delay or the occurrence of a deviant tone.

During the heartbeat discrimination task, an image of a heart was presented on the screen. This indicated that the participants should focus on their heartbeat. At the end of each trial, a response probe window was presented. The participants responded by pressing one of two assigned buttons to a yes/no probe whether the tone was simultaneous with their heartbeat or not. Immediately following the interoception, the participants evaluated their performance on a scale from one (random guessing) to five (100% certainty).

### Apparatus

ECG was measured with two Ag/AgCl electrodes placed on participants’ fifth left interclostal space at the midclavicular line and right sternum (ground electrode on participant’s right front side below ribs). The position of the electrode on sternum was adjusted so that a clear R-peak of the QWRS complex was detectable in the ECG signal, acquired using a specially designed device controlled by ARDUINO^®^ software. An in-house software script was used to detect individual R-peaks and to control the presentation of the heart-beat locked auditory stimuli. The device also sent a trigger marking each heartbeat to the MEG data, and the HR and HRV measures were calculated from these data using an in-house Python-based script.

### Variables

#### Interoceptive Sensitivity

Interoceptive sensitivity (IS) was measured as performance on a heartbeat detection task (discrimination task), calculated as the ratio of reports of simultaneity in all simultaneity trials. Even though the duration of the temporal delay in the simultaneity condition between the R-peak and the auditory stimulus was not quite the optimal for perceived simultaneity as observed in previous studies (approximately 200 ms; see, e.g., Wiens and Palmer, 2001), the correct response level the simultaneity trials was higher (0.64) than the chance level for binary yes–no responses (0.5) as confirmed by a one-sample *t*-test, *t*(49) = 4.22, *p* > 0.001, and higher than those studies reporting a similar test but using longer delays (e.g., Critchley et al., 2004; Garfinkel et al., 2015). In the non-simultaneity condition, the participant mean delays between heartbeat and auditory sound varied across participants between 250 and 530 ms (*M* ±*SD* = 381 ± 58 ms). These minimum and maximum delays happen to represent closely the delays of maximum and minimum perceived simultaneity (200 and 500 ms; e.g., Wiens and Palmer, 2001). For this reason, we could use the simultaneity condition only for measuring accuracy in the heartbeat discrimination task. The non-simultaneity condition was, nevertheless, used as a control condition for detecting biases in response behavior. Variable ‘ISE’ denotes self-evaluations of performance on the heartbeat discrimination task.

#### Physiological Measures

Heart rate was measured from ECG for each participant as the average interbeat (R-peak to R-peak) interval (IBI) during all trials in each condition. IBIs 50% longer or shorter than the mean of adjacent IBIs were rejected to correct for unregistered heartbeats in the ECG data. Heart rate variability (HRV) was measured as the standard deviation of IBIs in each condition for each participant (Fairclough and Goodwin, 2007). BMI was also calculated for each participant, as it has been observed to affect IS (e.g., Herbert and Pollatos, 2014).

#### Questionnaires

Individual temperament was measured with four scales:

(1) The Adult Temperament Questionnaire (ATQ) (Evans and Rothbart, 2007, Finnish version, translated by Katri Räikkönen-Talvitie and the Developmental Psychology Research Group of University of Helsinki; for validation in Finnish population, see Hirvonen et al., 2017). ATQ is a 77-item self-report questionnaire for adults measuring the general constructs or ‘factor scales’ Effortful control (ATQ-EC), Extraversion/Introversion (ATQ-EI), Negative affect (ATQ-NA), and Orienting sensitivity (ATQ-OS), all measured on a 1–4 Likert scale.
(2) The Karolinska Scales of Personality (KSP; Schalling et al., 1983; for the Finnish version, see, Af Klinteberg et al., 1990), KSP is a 130-item, 4-point Likert-type scale designed to measure biologically based personality constructs Extraversion/Introversion (KSP-EI), Anxiety (KSP-ANX), Conscientiousness (KSP-CON) and Aggression (KSP-AGG).
(3) The Reduced Emotional Intensity Scale (EIS-R, a brief version of the Emotional Intensity Scale [EIS], Bachorowski and Braaten, 1994). EIS-R, is a 17-item, 5-point Likert-type scale designed to measure emotional intensity. It consists of two highly intercorrelated subscales measuring the intensity of positive and negative emotions. Because of high intercorrelation (*r* = 0.84), only the total scores (EIS-R) of the questionnaire are used in the analyses.
(4) BIS/BAS, self-report scale measures responsiveness of the two general motivational system, the behavioral inhibition system (BIS) and approach system (BAS), related to approach and avoidance motivation, respectively (Carver and White, 1994). BIS/BAS is 21-item, 4-point Likert-type scale. It is divided to one BIS-related (BIS) and three empirically emerged BAS-related scales, but, not to complicate the analyses, the BAS-related ones were combined as one (BAS), according to the original design of the questionnaire.

### Data-Analysis

Correlation analysis. Pearson’s *r* was calculated between heartbeat detection scores, physiological and demographic data, as well as questionnaire scores. Multiple regression analyses were conducted to test which individual anxiety-related temperamental traits most strongly explain IS. Missing data were handled by substituting them with participant or scale mean, respectively, where applicable. An alpha level of 0.05 was used in all analyses.

## Results

### Descriptive Statistics and Correlations of Interoceptive Sensitivity to Temperament

Descriptive statistics for all variables, including Pearson’s *r* between all the reported variables, are given in **Table 1**.

**Table 1:** Correlation coefficients (Pearson’s *r*) and descriptive statistics (*n*, mean, standard deviation, skewness and kurtosis).

Variables	IS	ISE	IBI	HRV	BMI	ATQ_ EC	ATQ_ NA	ATQ_ EI	ATQ_ OS	KSP_ EI	KSP_ ANX	KSP_ CON	KSP_ AGG	BAS	BIS	EIS_ R	*n*	*M*	*SD*	Skewness	Kurtosis
IS	1.00																50	0.65	0.25	-0.52	-0.02
ISE	-0.07	1.00															49	3.26	0.84	-0.08	0.43
IBI	0.35^∗^	0.01	1.00														50	0.94	0.15	0.15	0.84
HRV	0.17	0.16	0.39^∗∗^	1.00													50	0.06	0.03	1.18	1.97
BMI	-0.11	0.10	-0.03	0.04	1.00												50	22.28	2.14	0.58	1.12
ATQ_ EC	-0.22	-0.01	-0.08	-0.12	-0.02	1.00											50	84.84	13.46	0.45	0.77
ATQ_ NA	0.29^∗^	0.23	0.04	0.03	-0.30^∗^	-0.53^∗∗^	1.00										50	94.10	19.39	-0.33	-0.65
ATQ_ EI	-0.35^∗^	-0.29^∗^	-0.11	-0.22	0.17	0.15	-0.39^∗∗^	1.00									50	79.90	12.30	-0.21	-0.56
ATQ_ OS	-0.06	0.29^∗^	-0.12	0.03	-0.19	-0.21	0.45^∗∗^	-0.11	1.00								50	71.40	13.34	-0.52	-0.50
KSP_ EI	-0.34^∗^	-0.10	-0.05	-0.21	0.12	-0.12	-0.25	0.78^∗∗^	0.02	1.00							50	76.84	10.16	0.09	-0.15
KSP_ ANX	0.34^∗^	-0.01	-0.10	0.04	-0.22	-0.61^∗∗^	0.65^∗∗^	-0.54^∗∗^	0.36^∗^	-0.38^∗∗^	1.00						50	104.99	23.80	0.59	0.31
KSP_ CON	-0.09	-0.27	-0.03	-0.16	0.01	0.46^∗∗^	-0.29^∗^	0.26	-0.39^∗∗^	-0.04	-0.52^∗∗^	1.00					50	93.54	10.56	-0.33	-0.86
KSP_ AGG	0.12	0.15	-0.02	0.10	0.08	-0.29^∗^	0.53^∗∗^	-0.31^∗∗^	0.43^∗∗^	-0.18	0.59^∗∗^	-0.53^∗∗^	1.00				50	53.20	8.76	0.30	-0.61
BAS	-0.10	0.06	-0.06	-0.17	-0.02	-0.07	0.14	0.42^∗∗^	0.23	0.61^∗∗^	-0.15	0.07	0.12	1.00			50	39.38	5.60	0.75	0.12
BIS	0.43^∗∗^	0.02	-0.16	-0.09	-30^∗^	-0.29^∗^	0.67^∗∗^	-0.57^∗∗^	0.28^∗^	-0.53^∗∗^	0.77^∗∗^	-0.19	0.44^∗∗^	-0.14	1.00		50	19.10	4.53	-0.25	-0.23
EIS_ R	0.35^∗^	0.01	-0.20	-0.16	-30^∗^	-0.16	0.55^∗∗^	-0.37^∗^	0.23	-0.30^∗^	0.55^∗∗^	-0.17	0.36^∗^	-0.09	0.64^∗∗^	1.00	50	100.05	10.10	-0.16	0.21


*^∗^*p* < 0.05, ^∗∗^*p* < 0.01.*

We observed a significant negative correlation of IS to heart rate, *r*(50) = -0.353, *p* = 0.012. Of the anxiety-related temperamental traits, IS was found to be positively associated with ATQ-EI, KSP-EI, KSP-ANX, ATQ-NA, EIS-R, and BIS. Subjective evaluations of the performance or BMI did not correlate to the actual performance on the heartbeat detection task.

### Regression Analysis

To investigate further the relation between IS and temperament, a multiple linear regression analysis with IS as independent variable. We selected the theoretically least redundant traits that were significantly associated with IS: ATQ-EI, KSP-ANX, EIS-R and BIS as predictors. As these measures were intercorrelated (multicollinearity), variance inflation factors (VIFs) are also reported, and Backward elimination method (selection criteria for variables: *p* = 0.05 for entry, *p* = 0.1 for removal) was applied to select the variable(s) that best predict IS. **Table 2** shows the models, the first comprising of all entered variables, and the relevant statistics for all the models. The fourth and final model exhibits the best fit, excluding all the variables other than BIS. The Mallow’s Cp reached its lowest value, indicating the superiority of this model, and the multicollinearity was solved (VIF = 1). The regression analysis thus indicates that BIS alone can best predict IS, and the prediction potential of the other variables is probably due to shared variance with BIS.

**Table 2:** Regression analysis for interoceptive sensitivity (Standardized coefficients).

Variables	Models							
	1	VIF	2	VIF	3	VIF	4	VIF
ATQ-E/I	-0.17	1.52	0.16	1.46	0.16	1.46	–	–
KSP-ANX	-0.04	2.53	–	–	–	–	–	–
BIS	0.28	3.11	0.26	2.14	0.34^∗^	1.46	0.43^∗∗^	1.00
EIS-R	0.13	1.71	0.13	1.69	–	–	–	–
Mallow’s Cp	5.00		3.03		1.52		0.53	
Adjusted *R^2^*	0.14		0.16		0.17		0.17	


*^∗^*p* < 0.05, ^∗∗^*p* < 0.01.*

### Response Bias

Our measure for IS was based on heartbeat discrimination performance in the simultaneity condition but not in the non-simultaneity condition. It is therefore possible that, rather than differences in performance levels, some sort of response bias could explain the present results; e.g., extravert individuals have exhibited a lenient response style in previous studies (see, e.g., Koelega, 1992), which could have led to an excess of simultaneity reports (i.e., yes-responses to the simultaneity probe). However, the same should have been visible in the non-simultaneity condition, but no such tendency was observed (e.g., correlation of ATQ-EI to the amount of yes-responses in the heartbeat detection task: *r*[49] = -0.10, *p* = 0.48; BIS: *r*[49] = 0.08, *p* = 0.59). It seems highly improbable that any response style related to temperamental traits could have biased the results.

## Discussion

We explored the relations between IS and other individual constitutional traits in a normal healthy population. As expected based on previous research, IS was particularly correlated to measures of anxiety-related temperamental constructs. These included temperamental anxiety, negative affect, introversion and experienced emotional intensity. The temperamental trait behavioral inhibition predicted most strongly performance on the heartbeat detection task. Based on our present results from the regression analysis, the association with the other avoidance related measures is probably due to shared variance with temperamental behavioral inhibition. Compatibly, behavioral inhibition is theoretically seen as a predecessor of anxiety (Svihra and Katzman, 2004; Muris et al., 2011) and introversion (Shatz, 2005).

The increased sensitivity to one’s bodily milieu by behavioral inhibition may relate to differencial tendencies in allocation of attention in BIS sensitive individuals. Heightened IS presumably leads the individual to attend to his/her bodily signals more than low IS. This could, in turn, predispose the individual to enhanced bodily responsiveness in social interaction. Alternatively, the results may reflect differential, globally and spatially oriented cognitive control abilities specific to BIS sensitive individuals, as observed, e.g., by Prabhakaran et al. (2011) in Stroop tasks measuring these dimensions of selective attention. The heartbeat discrimination task, based on a comparison of sensory signals across sensory modalities, requires more global cognitive control compared to the more straightforward tracking task.

Interestingly, from a cortical perspective, the right insula, which mediates the relationship between bodily sensitivity and anxiety (Terasawa et al., 2013), is also critical for both IS (Pollatos et al., 2016) and multisensory simultaneity perception (Bushara et al., 2001). The interoceptive brain network regions in the right hemisphere have also been generally implicated in avoidance motivation (Craig, 2005, 2014). Perhaps this functional architecture also mediates the link between IS and behavioral inhibition/anxiety. Nonetheless, in adult humans, temperament is typically studied by questionnaires, the results of which only indirectly reflect possible underlying neurobiological systems.

Our results also showed an association between higher IS and lower heart rate, compatibly with previous studies using a simultaneity task (Knapp-Kline and Kline, 2005; Fairclough and Goodwin, 2007; Schulz et al., 2013). Other studies, where mostly the heartbeat tracking task was used, have either supported the opposite (Eichler and Katkin, 1994; Wiens et al., 2000; Herbert et al., 2007, 2010; Pollatos et al., 2007), or, no such relation was observed (e.g., Yoris et al., 2015). As the contribution of slow heart rate to the simultaneity discrimination has been systematically observed, it may be, as Knapp-Kline and Kline (2005) theorized, that slow heart rate allows the perceptual system more time to determine simultaneity. As distinct heartbeat detection tasks may bring forth distinct aspects of IS and its relation to individual traits, such as effects of stress (Schulz and Vögele, 2015), the use of a different task can yield differing results also for regarding individual traits and wellbeing. It is advisable to use multiple tasks, e.g., heartbeat tracking and detection tasks in future studies, as also described in the context of clinical symptoms (Khalsa and Lapidus, 2016).

We applied a heartbeat discrimination task, which reflects a specific, objective form of interoceptive awareness. Others, more subjective oriented forms have been proposed (Mehling et al., 2012; Garfinkel et al., 2015; Ceunen et al., 2016). It would be interesting to compare results from studies using subjective constructs to the present one. Such studies would deepen our understanding of how both subjective and objective body awareness mesh with other individual traits.

The present results are based on background variables of a more extensive brain imaging study, which forced to use a relatively small sample size (*n* = 50). The results should thus be seen as tentative. However, the observed associations between IS and temperamental variables were strong, and they have been observed previously in studies using similar sample sizes (e.g., Pollatos et al., 2007, *n* = 38; Herbert et al., 2007, *n* = 37). The measured variables were also theoretically convergent, which adds to the reliability of the results.

All in all, our results suggest that individual differences in interoception are core constituents of a human individual’s personality, together with other constitutional traits such as temperament. The interoceptive system plays a critical role in the individual’s subjective experience of health and wellbeing – as well as perception of his/her relation to the immediate environment. Correspondingly, IS determines how strongly and in what way an individual responds to threats to one’s wellbeing. High IS has previously been linked with individual traits, such as anxiety, especially in clinical context with an acutely negative influence on the subjective feeling of wellbeing. On the other hand, high IS seems to equip an individual with a unique coping capacity of bodily and social disturbances (Füstos et al., 2013; Pollatos et al., 2015). Future studies are needed to solve the issue whether IS is a predisposing factor, a concomitant factor, or a result of coping with, wellbeing together with other individual traits.

## Author Contributions

PL analyzed the data, participated in interpretation of the results, and prepared the initial version of the manuscript. TP conceived the study, designed the experimental setup, supervised data collection, provided critical comments for data analysis, and participated in interpretation of the results and in preparation of the manuscript.

## Conflict of Interest Statement

The authors declare that the research was conducted in the absence of any commercial or financial relationships that could be construed as a potential conflict of interest.
